# Leveraging Electronic Health Records Data for Enhanced Colorectal Cancer Screening Efforts

**DOI:** 10.13023/jah.0204.07

**Published:** 2020-09-01

**Authors:** Adam Baus, Lauren E. Wright, Stephenie Kennedy-Rea, Mary E Conn, Susan Eason, Dannell Boatman, Cecil Pollard, Andrea Calkins, Divya Gadde

**Affiliations:** West Virginia University School of Public Health; West Virginia University Cancer Institute; West Virginia University School of Public Health

**Keywords:** Appalachia, electronic health records data, cancer screening, quality improvement, rural

## Abstract

**Introduction:**

Colorectal cancer is the third most common type of cancer in the United States for men and women combined. While the current threat of disease nationally is significant, the majority of colorectal cancer cases and deaths could be prevented through established screening tests and guidelines. Within the Appalachian region and West Virginia in particular, colorectal cancer is a significant public health problem. A more systematic, comprehensive approach to preventing and controlling cancer is essential.

**Methods:**

Through the West Virginia Program to Increase Colorectal Cancer Screening, primary care systems across the state received data-informed practice facilitation designed to increase screening rates.

**Results:**

Year-1 cohort health systems had an overall baseline screening rate of 28.4% during calendar year 2014. This rate increased and remained steady during the three follow-up measurement time periods, with a rate of 49.5% during calendar year 2018. This increase is notably greater than comparable health systems not part of the initiative.

**Implications:**

Lessons learned in increasing colorectal cancer screening rates are applicable to other priority health needs as well.

## INTRODUCTION

Colorectal cancer (CRC) is the third most common type of cancer in the U.S. for men and women combined.^1^ In 2016, the most recent year for which national data on CRC incidence are available, 141,270 new cases of CRC were reported with 52,286 people dying from this condition.^2^ While the current threat of disease nationally is significant, the majority of CRC cases and deaths could be prevented through knowledge of cancer prevention and established screening tests and guidelines.^1^ The Centers for Disease Control and Prevention (CDC) estimates that approximately nine of every ten people whose CRC is found early and treated appropriately are alive 5 years after the initial diagnosis.^2^ Additionally, between 50% and 60% of CRC deaths could be eliminated through regular screening. Within the Appalachian Region, and West Virginia (WV) in particular, CRC is an especially significant public health problem. WV is the only state located solely within Appalachia, a heterogeneous and economically disadvantaged portion of the U.S.^3^ WV has elevated CRC mortality and incidence rates when compared with the U.S. overall. In 2016, for every 100,000 people, 37 new cases of CRC and 14 deaths were reported in the U.S., and 43 new CRC cases and 17.7 deaths were reported in WV.^4^ The low rate of CRC cases found in the local disease state (39%) suggests that there is a depressed level of CRC screening in the region.^5^ Cultural, social, and physical barriers can have an impact on an individual’s willingness to be screened.^6^ Residents in rural states, like WV, face additional socioeconomic barriers to care that can make screening more challenging. Access to appropriate medical care is another barrier, as many rural communities are classified as Medically Underserved Areas and/or Health Professional Shortage Areas.

Recent data from the West Virginia Cancer Registry highlights the complex, diverse nature of cancers in the state and the need for strategic efforts in prevention and early detection. Essential is the need for WV primary care to establish a more systematic, comprehensive approach to preventing and controlling cancer. In response to the substantial health crisis facing the state, the West Virginia University Cancer Institute (WVUCI), Cancer Prevention and Control (CPC) applied to the CDC’s Colorectal Cancer Control Programs (CRCCP) initiative for funding. In June 2015, the application for funding was approved for a 5-year period, and the WV Program to Increase Colorectal Cancer Screening (WVPICCS) was created.

The WV Program to Increase Colorectal Cancer Screening works by utilizing U.S. Preventive Services Task Force–recommended evidence-based interventions shown to increase CRC screening rates. Interventions include provider assessment and feedback, reducing structural barriers, provider reminder and recall systems, and client reminder systems. In addition, using the Task Force on Community Preventive Services recommendations, partner clinics may choose to work with WVPICCS on supportive activities such as small media and patient navigation. WVPICCS utilizes a practice-change model to partner with primary care practices across WV to make systems-based changes using these evidence-based interventions. It is through the implementation of these strategies that consistent increases in CRC screening rates are expected. The goal is to increase CRC screening in partnering primary care settings by at least 10% from baseline, working toward the national goal of 80%. These interventions, designed to better equip primary care with knowledge, skills, and abilities to sustain systems-level change, require enhanced health informatics technology (HIT) skills in using electronic health records (EHR) data for cancer screening.

The integration of EHRs into primary care practices and hospitals is an acknowledged tool to improve healthcare decisions and patient outcomes.^7^ EHRs can strategically identify and monitor patients for specific services.^8^ This includes identification of patients eligible for CRC screening, facilitation of reminder and recall systems, and monitoring of referrals, delivery, and outcomes for quality and performance measurement purposes.^8^ These functions align with research that suggests that reminder systems, feedback, and audits are successful tools to increase CRC screening rates.^8^ In 2009, Congress adopted the Health Information Technology for Economic and Clinical Health Act (HITECH), which focuses EHR use to improve patient care through Meaningful Use.^7^ While the financial incentives behind Meaningful Use have increased the adoption of EHR systems, there have been significant challenges to this transition for many primary care practices and hospitals.

Challenges include a lack of knowledge about best practices for implementation and no incentives for integration and collaboration.^9^ In addition, primary care practices and hospitals that serve safety-net populations often fall behind their peers during EHR implementation.^9^ Practice challenges such as provider/staff engagement, clarity on EHR vendor selection, and clinic workflow adaptation are cited as key difficulties for EHR implementation.^10^ Historic challenges in tracking clinical measures have caused some primary care practices to adopt work around procedures that detract from the time-saving benefits of EHRs.^11^,^12^ Without resolution of these challenges, primary care practices and hospitals are not able to experience the benefits of better patient outcomes and improved healthcare decisions associated with successful EHR utilization. To combat these challenges and to enhance program sustainability, WVPICCS utilized the expertise of HIT specialists to improve clinic EHR use and data reporting. These individuals assessed clinic capacity to leverage EHR tools and underlying data, assisted in the review and evaluation of outcomes data, and provided overall encouragement and guidance in developing continuous quality-improvement cycles designed to improve clinic workflows and data capture.

This study presents an analysis of change in CRC screening rates among the six health systems included in the Year-1 WVPICCS cohort. These six health systems represent 16 individual primary care clinics. Changes in rates are placed in context of targeted analytics and practice facilitation support in evaluating EHR data quality, modifying office procedures to address challenges, and overall improved application of clinical data to patient navigation and population health efforts. Analysis of program evaluation data was reviewed and deemed nonhuman subjects research by the West Virginia University Institutional Review Board, Protocol # 1907654102.

## METHODS

Prior to the start of implementation of evidence-based strategies within each partner clinic, WVPICCS staff conducted an initial site visit with administration and key informants to better understand the practice structure and specific clinic needs. Each partner clinic provided WVPICCS with an initial baseline data report that showed their screening rates and patient demographics for the previous year. This information assisted program staff as they developed implementation plans tailored for each clinic.

**Figure 1:**
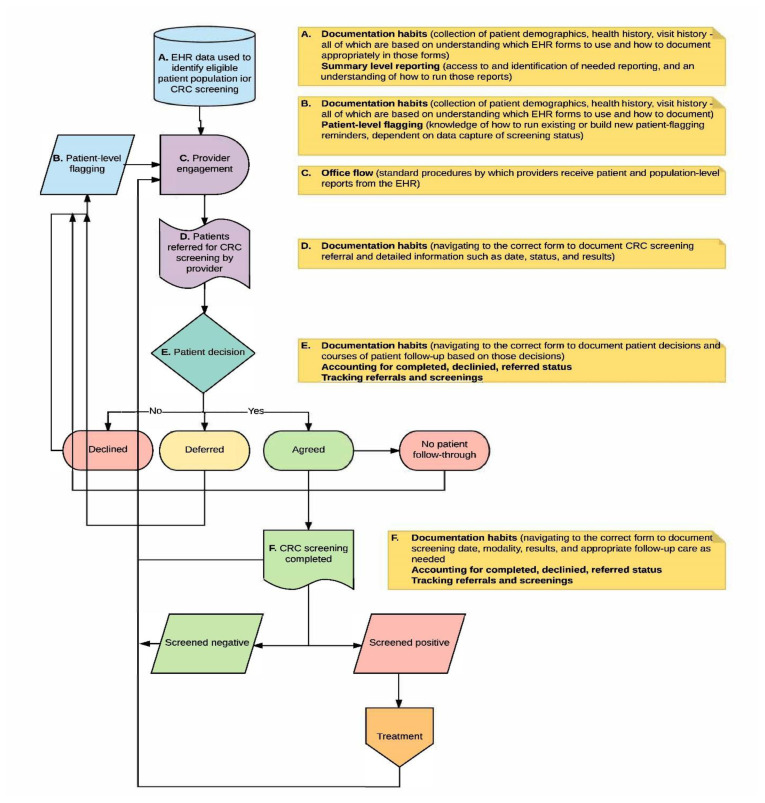
Colorectal Cancer Screening Best Practices: EHR Training Areas for WVPICCS Partners Diagram for mapping EHR data flow, used for identifying issues in data collection and data completeness.

A key component of the WVPICCS program is related to EHR integration. Prior to implementation, each partner clinic participated in a Health Information Technology (HIT) Assessment. A HIT Specialist, part of the WVPICCS team, visited each clinic to assess the capabilities and challenges they faced with their EHR system. Partner clinics were then presented with a report of the HIT findings and encouraged to work with their EHR vendor and the WVPICCS HIT Specialist to develop solutions to challenges faced. The WVPICCS HIT Specialist provided specific, ongoing EHR support and training throughout the duration of the 2-year project. Support was targeted to the development of best practices in using the EHR for CRC screening and prevention, focused on identifying and addressing challenges via targeted training and technical assistance (Figure 1).Technical assistance was provided in a combination of in-person, web-based, and telephone-based delivery. Support sessions were not only planned, but available ad-hoc at the request of partner clinics.

Targeted healthcare team members at each health system received customized HIT support via the WVPICCS program. Efforts focused, foremost, on addressing the lack of standardization in data entry into the EHRs given the impact of this factor on data quality. Process mapping was used to better understand the people involved and steps needed to ensure accurate CRC screening rates (Figure 1). Through measurement of ongoing rates at the clinic and provider levels, screening rates were monitored over time to evaluate change. Concurrent to this, participating health systems received additional support from WVPICCS staff in integrating provider-level and site-level report cards into monthly project meetings. Through the entire effort, supplemental analytics and reporting support were provided to these sites, with the intent on increasing the in-house skill sets and knowledge base of the health system partners. The goal was sustainable change and better use of data analytics to guide practice once technical assistance decreased. There was a constant focus to balance technical assistance without creating nonsustainable dependencies.

## RESULTS

Health Information Technology assessments with the Year-1 WVPICCS health systems revealed clear commonalities in strengths and challenges in EHR use. Overall, these health systems demonstrated long-term commitment to improving quality of care and patient outcomes. Commitment was identified through a history of participation in a variety of state- and national-level quality-of-care improvement efforts, leaning collaboratives, and achievement of Patient Centered Medical Home recognition through the National Committee for Quality Assurance. However, the HIT assessments and follow-up discussions also revealed common challenges in fully integrating EHRs into patient and population health improvement. Table 1 presents summary findings from Year-1 HIT assessments, organized by four prominent themes. These themes served as a guide in helping to transform these challenges into issues capable of being addressed and ameliorated over time. First, each of the 6 health systems (100.0%) expressed concern over the need to better standardize the way in which members of the healthcare team entered EHR data. The lack of standardized procedures was a consistent barrier to systems improvement. Second, half of these health systems (50%) expressed a lack of technical assistance and training from their EHR vendors. This dearth of support tended to result in health system partners relying primarily on experiential learning only, resulting in inconsistencies and decreased data quality. Third, half of the Year-1 cohort partners (50%) expressed a lack of data tracking, reporting, and analytics functionality within their EHRs. These perceived systems limitations tended to frustrate healthcare team members in light of an already present sense of lacking EHR vendor support. Fourth, half of these health systems (50%) noted that some of the more significant analytics tools within their EHRs, such as provider reminders and patient recall features, were underutilized. This issue was, at times, treated as a by-product of the lack of confidence in their EHR data quality —an issue itself related to nonstandard data entry and lacking vendor-driven training.

**Figure 2:**
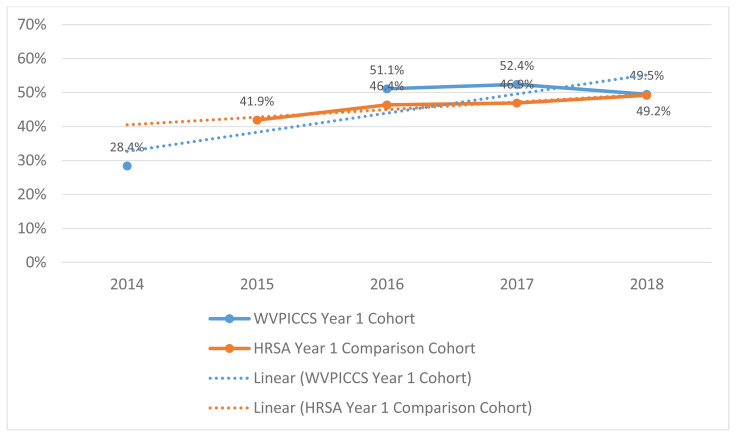
Comparison of Colorectal Cancer Screening Rates of WVPICCS FQHCs in Year 1 Cohort Compared to non-WVPICCS FQHCs Trend lines showing aggregate values for WVPICCS FQHCs in Year-1 Cohort compared to non-WVPICCS FQHCs. Baseline rates are shown for 2014. Data are not available for 2015. HRSA data are displayed for non-PICCS FQHCs for the reporting period. HRSA regression equation: Screening rate = 0.0393433*Year + −78.8834 (P=0.005). WVPICCS regression equation: Screening rate = 0.0562604*Year + −112.982 (P<0.001).

The Year-1 cohort CRC screening rates increased substantially over time. WVPICCS Year-1 cohort sites had an overall baseline screening rate of 28.4% of patients aged 50–75 years receiving guideline-based CRC screening during calendar year 2014. This rate increased and remained steady during the three follow-up measurement time periods, with a rate of 49.5% during calendar year 2018. This increased screening rate in WVPICCS participating health systems was examined relative to federally qualified health centers not engaged in the program and found a notable difference. Specifically, CRC screening rates increased at a more significant rate (P<0.001) during the measurement period as compared to those health systems not taking part in the initiative (P=0.005) (Figure 2). Future work within WVPICCS will aim to better position the intervention impacts to be attributable to more specific components of the HIT and practice facilitation processes.

## IMPLICATIONS

The WVPICCS initiative shows that combining a practice facilitation model with in-depth, targeted HIT support addresses long-standing issues in using EHRs for cancer screening and prevention. A hands-on, collaborative approach to learning from and working with primary care proves effective in increasing screening rates in a way which is sustainable and beneficial to health systems and patients served. The approach used in this effort has application beyond CRC screening specifically. Better understanding data collection and flow in primary care, limitations of EHRs, skill-sets and comfort levels of primary care members, and overall trust in data are paramount to any quality of care improvement effort designed to measure and positively affect health outcomes.

**SUMMARY BOX**

**What is already known about this topic?** While low colorectal cancer screening rates are a problem nationally, this problem is even more significant in rural, Appalachian states such as West Virginia.

**What is added by this report?** This report addresses the issues surrounding EHR integration in primary care clinics and offers a successful and sustainable solution to these issues by having EHR HIT specialists play a key role in the implementation of primary care-based programs.

**What are the implications for future research?** Low colorectal cancer screening rates in Appalachian states can be ameliorated through HIT training coupled with data-informed practice facilitation in primary care aimed at increasing the knowledge, skills, and ability of primary care to better leverage HIT tools and clinical data for enhanced patient navigation and population health efforts.

## Figures and Tables

**Table 1:** Summary of Findings from Health Information Technology Assessments with Year-1 Cohort WVPICCS Health Systems

Category	N	% of Total
Need for standard operating procedures for EHR data documentation		
Yes	6	100.0
Limited support from EHR vendor		
Yes	3	50.0
No	3	50.0
EHR limitations in data tracking, reporting, analysis		
Yes	3	50.0
No	3	50.0
EHR features going underutilized		
Yes	3	50.0
No	3	50.0

EHR, electronic health records

## References

[1] Centers for Disease Control and Prevention Basic information about colorectal cancer 2020 http://www.cdc.gov/cancer/colorectal/basic_info/index.htm

[2] Curry WJ Lengerich EJ Kluhsman BC Academic detailing to increase colorectal cancer screening by primary care practices in Appalachian Pennsylvania BMC Health Serv Res 2011 11 112 10.1186/1472-6963-11-112. 21600059 PMC3128846

[3] Knight JR Kanotra S Siameh S Jones J Thompson B Thomas-Cox S Understanding barriers to colorectal cancer screening in Kentucky Prev Chronic Dis 2015 2015 12 E95 10.5888/pcd12.140586. PMC4473604 26086608

[4] Centers for Disease Control and Prevention and National Cancer Institute US Cancer Statistics Working Group. US cancer statistics data visualizations tool 2020 https://gis.cdc.gov/Cancer/USCS/DataViz.html

[5] West Virginia Department of Health and Human Resources and West Virginia University Cancer Institute 2019 West Virginia cancer burden report https://oeps.wv.gov/cancer/documents/data/burdenreport2019.pdf

[6] Blumenthal D Tavenner M The “meaningful use” regulation for electronic health records N Engl J Med 2010 363 501 4 10.1056/NEJMp1006114. 20647183

[7] Klabunde CN Lanier D Breslau ES Improving colorectal cancer screening in primary care practice: innovative strategies and future directions J Gen Intern Med 2007 22 8 1195 1205 10.1007/s11606-007-0231-3. 17534688 PMC2305744

[8] Ashish KJ Meaningful use of electronic health records: the road ahead JAMA 2010 304 15 1709 10 10.1001/jama.2010.1497 20959581

[9] Heisey-Grove D Danehy LN Consolazio M Lynch K Mostashari F A national study of challenges to electronic health record adoption and meaningful use Med Care 2014 52 2 144 8 10.1097/MLR.0000000000000038. 24309669

[10] O’Malley AS Draper K Gourevitch R Cross DA Scholle SH Electronic health records and support for primary care teamwork J Am Med Inform Assoc 2015 22 2 426 34 10.1093/jamia/ocu029 25627278 PMC4394968

[11] Vishwanath A Singh S Winkelstein P The impact of electronic medical record systems on outpatient workflows: a longitudinal evaluation of its workflow effects Int J Med Inform 2010 79 778 91 10.1016/j.ijmedinf.2010.09.006. 20947415

[12] Baron R Quality improvement with an electronic health record: achievable, but not automatic Ann Intern Med 2007 147 8 549 52 10.7326/0003-4819-147-8-200710160-00007. 17938393

